# Resistance Switching in Polycrystalline C12A7 Electride

**DOI:** 10.3390/mi13111917

**Published:** 2022-11-06

**Authors:** Ivan D. Yushkov, Gennadiy N. Kamaev, Vladimir A. Volodin, Pavel V. Geydt, Aleksandr V. Kapishnikov, Alexander M. Volodin

**Affiliations:** 1 Laboratory of Functional Diagnostics of Low-Dimensional Structures for Nanoelectronics, Department of Physics, Novosibirsk State University, Pirogova Str., 2, 630090 Novosibirsk, Russia; 2 Rzhanov Institute of Semiconductor Physics, Siberian Branch of the Russian Academy of Sciences, Lavrentyev Ave. 13, 630090 Novosibirsk, Russia; 3 Federal Research Center Boreskov Institute of Catalysis, Siberian Branch of the Russian Academy of Sciences, Prospekt Lavrentieva, 5, 630090 Novosibirsk, Russia

**Keywords:** polycrystalline mayenite, C12A7, electride, memristor, temperature dependence of conductivity

## Abstract

The memory (memristive) properties of an electride material based on polycrystalline mayenite (C12A7:e^−^) were studied. The phase composition of the material has been confirmed by such methods as XRD, TEM, Raman, and infrared spectroscopy. The electride state was confirmed by conductivity measurements and EPR using a characteristic signal from F^+^—like centers, but the peak at 186 cm^−1^, corresponding to an electride with free electrons, was not observed explicitly in the Raman spectra. The temperature dependence of current–voltage characteristics in states with low and high resistance (LRS and HRS) has been studied. In the LRS state, the temperature dependence of the current has a non-Arrhenius character and is described by the Hurd quantum tunnelling model with a Berthelot temperature of 262 K, while in the HRS state, it can be described in terms of the Arrhenius model. In the latter case, the existence of two conduction regions, “impurity” and “intrinsic”, with corresponding activation energies of 25.5 and 40.6 meV, was assumed. The difference in conduction mechanisms is most likely associated with a change in the concentration of free electrons.

## 1. Introduction

Resistive random-access memory (ReRAM) is one of the most relevant objects of research in memory community because this type of device can be used as a non-volatile memory device. ReRAM has high speed [1] and good storage time compared to older generation storage devices. ReRAM uses the effect of the so-called controlled breakdown, in which the metal–insulator–semiconductor (MIS) or metal–insulator–metal (MIM) structure, the dielectric, called the active layer, is capable of abruptly changing conductivity upon reaching a certain value of externally supplied tension [2]. This is due to phase or structural changes in the active layer. At the moment, several mechanisms explain the resistive switching in ReRAM [3]. However, discussions on the transport mechanism are currently ongoing. This is due to the complexity of the structures in which the memristor effect was found.

The resistive switching effect has been found In many non-stoichiometric oxides and nitrides of metals and semiconductors, such as TiO_x_, SiO_x_, SiN_x_, GeO_x_, GeSi_x_O_y_, HfO_x_, etc. [4,5,6,7,8]. However, in such structures, during operation, the active medium is destroyed, which can lead to the uncontrolled degradation of the characteristics required for storage devices. In this regard, there are problems with the creation of ReRAM based on these materials.

Mayenite-type calcium aluminate, which is complex oxide 12CaO·7Al_2_O_3_ (also known as C12A7), is interesting in that its properties can be easily changed by varying the composition of the anionic sublattice and replacing oxygen anions with anions of a different type. In this case, the [Ca_24_Al_28_O_64_]^4+^ cationic framework remains unchanged [9,10,11,12]. Thus, by varying the oxygen stoichiometry (δ = 0–1 for Ca_12_Al_14_O_33−δ_) in mayenite, it is possible to form electride state C12A7:e^−^, in which the role of anions is played by electrons [12,13] and significantly affects the electrical properties. For the first time, this state for mayenite was described in the works of the H. Hosono group about 20 years ago [10,11]. Several publications have demonstrated the possibility of using an inorganic electride based on this compound as a material to create components of electronic devices due to the presence of several resistive states [13,14]. To reduce costs and simplify the production of electronic devices based on C12A7:e^−^, it is more relevant to synthesize and study this substance in the polycrystalline state of a substance, which can impose several features on the properties of the substance, unlike a monocrystalline, due to physicochemical interactions on intergrain boundaries of matter, for example. Broad categories of modern materials, such as thin films, whiskers, or aerogels [15,16,17,18], involve the use of a compound in a polycrystalline or nanocrystalline state.

The possibility of memristor switching in polycrystalline C12A7:–^−^ has already been shown [13]. The purpose of this work is to conduct a detailed study on the current–voltage characteristics (I–V curves) of this material based on complex oxide C12A7, which has memristor properties. In particular, the study of the temperature dependence of conductivity in a low-resistance state (LRS) and a high-resistance state (HRS) (which are occasionally found in the literature as an ON state and an OFF state, correspondingly) was carried out.

## 2. Fabrication Technique and Characterization Methods

**Figure 1:**
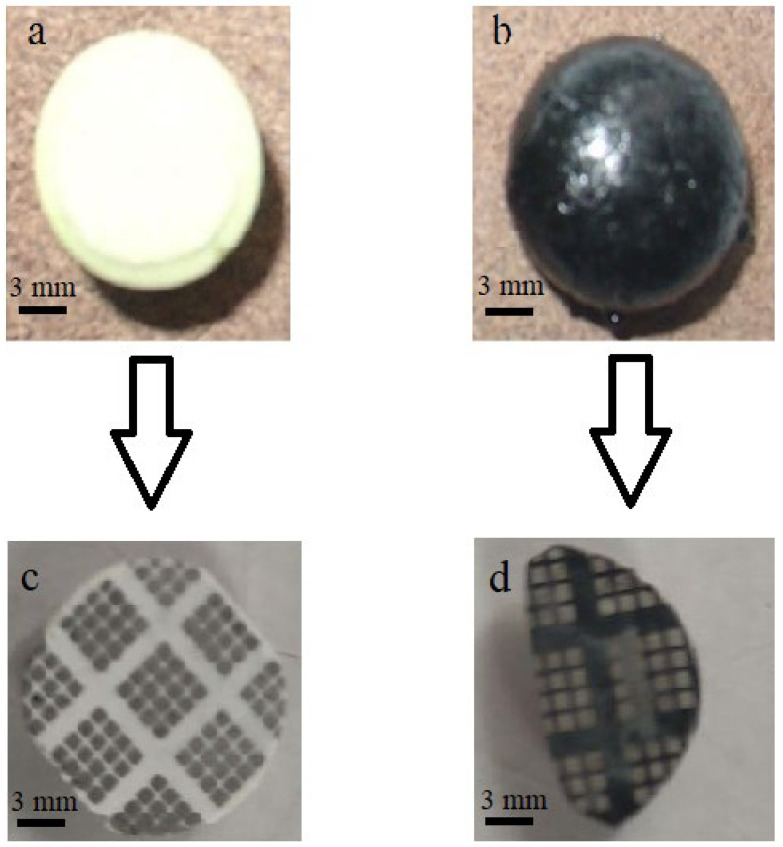
Photo of samples C12A7:O^2−^ (**a**,**c**) and C12A7:e^−^ (**b**,**d**) made before (**a**,**b**) and after (**c**,**d**) deposition of nickel contacts.

In this work, the method of synthesis of mayenite previously described in [13,19,20] was used. Aluminum hydroxide (Condea Pural SB—1, pseudo–boehmite, >99.9%) and calcium carbonate (Reachim, >99.97%) were used as starting materials for the synthesis. The first stage of the synthesis was to obtain CaO by decomposing CaCO_3_ in a muffle furnace in the air at 950 °C for 6 h. After that, the resulting CaO was added to a suspension of aluminum hydroxide in distilled water at room temperature with continuous stirring. The final ratio of the components corresponded to the stoichiometry of mayenite (12CaO·7Al_2_O_3_). The mixture was thoroughly stirred in distilled water for 10 h, filtered, and dried at 110 °C. Then, it was calcined in a muffle furnace in the air at 500 °C for 6 h. The resulting sample was heated in an Ar flow at a rate of 3 °C/min, followed by holding at 1360 °C (C12A7:O^2−^, Figure 1a) and 1450 °C (C12A7:e^−^, Figure 1b) for 6 h. 

To conduct electrophysical experiments, disks 1 mm thick were cut from the obtained samples, on which contacts were applied (Figure 1c,d).

To carry out structural studies by powder X-ray diffraction (XRD) and to measure the electron paramagnetic resonance (EPR) spectra, the obtained samples were ground in an agate mortar. Powders of fraction d < 0.25 mm were used for measurements. Before measurement by electron microscopy, the sample was dispersed using an ultrasonic disperser and put to a copper grid.

Powder XRD studies were carried out on an ARL X’tra diffractometer (ThermoFisher Scientific, USA) with a Cu–Ka radiation source (λ = 1.5418 Å). The diffraction patterns were measured in the range 2θ = 15–85°, the step was 0.05, and the signal accumulation duration was 3 s for each point. The processing of diffraction patterns and semi-quantitative phase analysis was carried out by refining the complete profile by the Rietveld method in the GSAS–II program [21].

The EPR spectra were recorded at room temperature using an ERS–221 spectrometer (Center of Scientific Instruments Engineering, Leipzig, German Democratic Republic) operating in the X-band. The concentrations of the paramagnetic species were determined by numerical double integration with baseline compensation. A dark-colored, crystalline powder 2,2–diphenyl–1–picrylhydrazyl (DPPH), as the primary standard for quantitative EPR spectrometry, was used for the calibration of the spectrometer before the study.

High-resolution transmission electron microscopy (HRTEM) images were obtained on a JEM–2200FS (JEOL, Japan) transmission electron microscope with a spherical aberration corrector at an accelerating voltage of 200 kV.

The vibrational properties of the materials were studied using Raman spectroscopy. The Raman spectra were recorded at room temperature in the backscattering geometry; a fiber laser with a wavelength of 514.5 nm and power of 100 mW was used for excitation. A T64000 spectrometer (Horiba Jobin Yvon, France) was used. The spectral resolution was no worse than 2 cm^−1^. A silicon matrix of photodetectors cooled with liquid nitrogen was used as a detector. An attachment for microscopic studies of Raman scattering was used. The power of the laser beam reaching the sample was 2 mW. An analysis of scattered light polarization was not carried out. To minimize the heating of the structures under the laser beam, the sample was placed slightly below the focus and the spot size was 10 microns. All used analytical equipment belongs to the sector of Optical Methods for the Study of Nanostructured Materials of the Analytical and Technological Research Center (ATRC) “High Technologies and New Materials” of Novosibirsk State University.

The vibrational spectrum of the mayenites was also studied using Fourier–transform infrared (FTIR) spectroscopy. The FT–801 Fourier spectrometer (SIMEX, Novosibirsk, Russia) was used to register FTIR reflectance spectra. The spectral range of the device was from 500 to 4000 reciprocal centimeters. The resolution was, depending on the speed and amplitude of the scan, from 0.5 to 4 cm^−1^. In our case, we used a resolution of 4 cm^−1^. Due to the opacity (due to scattering on inhomogeneities in polycrystalline grains), it was impossible to study the samples under study in transmission geometry. Therefore, the reflection spectra were studied using the specular reflection setup. A metal mirror was used as a reference sample, and the reflection coefficient from this sample did not contain features in the studied region of the spectrum.

The study of the electrophysical characteristics of the obtained samples was carried out on metal–mayenite–metal structures. Top electrodes made of nickel 200 nm thick with a contact area of 0.5 mm^2^ were deposited through a mask by magnetron sputtering in an Ar atmosphere. A continuous nickel layer of the same thickness was deposited on the bottom side. Measurements of the I–V curve (voltage–current characteristics) of the obtained structures and the study of the operation stability of the C12A7:e^−^ sample were carried out at room temperature using a B2902A two-channel source meter (Agilent, USA) with micropositioners connected to it with probes. The temperature characteristics of the conductivity and I–V curves at different temperatures were carried out using a Keithley 2400 m source (Keithley, USA) with a Linkam LTSE420–PB4 stage (Linkam Scientific Instruments, United Kingdom) connected to it. The temperature change was carried out using a Linkam T95 temperature controller and a Linkam LNP 95 liquid nitrogen pump. Measurements of current–voltage characteristics at various temperatures were carried out at a voltage of 0.5 V. The initial temperature was 300 K. The sample was in the LRS state at the initial moment of the sweep. With decreasing temperature, the current decreased, hence the conductivity decreased. After reaching a temperature of 100 K, the sample switched to the HRS state, a voltage of 0.5 V was set at which measurements were made, and heating was carried out to 300 K. The recording of additional I–V curves at extreme temperature points was carried out by analogy with registration at room temperature. The voltage sweep for 100 K was taken from 6 to −5 V.

## 3. Results

### 3.1. XRD and TEM Studies

The studied samples based on polycrystalline mayenite are shown in Figure 1. A white sample in Figure 1a contains oxygen anions in the unit cell of the crystal structure, as indicated by the color of the sample and the low specific conductivity σ ≈ 10^−16^ Om^−1^·cm^−1^. In the case of the mayenite sample in the electride state (Figure 1b), this is represented by the black color of the sample and high conductivity σ ≈ 10^−11^ Om^−1^·cm^−1^. To study the current–voltage characteristics, a layer of nickel 200 nm thick was deposited on both sides of the disks 1–2 mm thick (Figure 1c,d).

**Figure 2:**
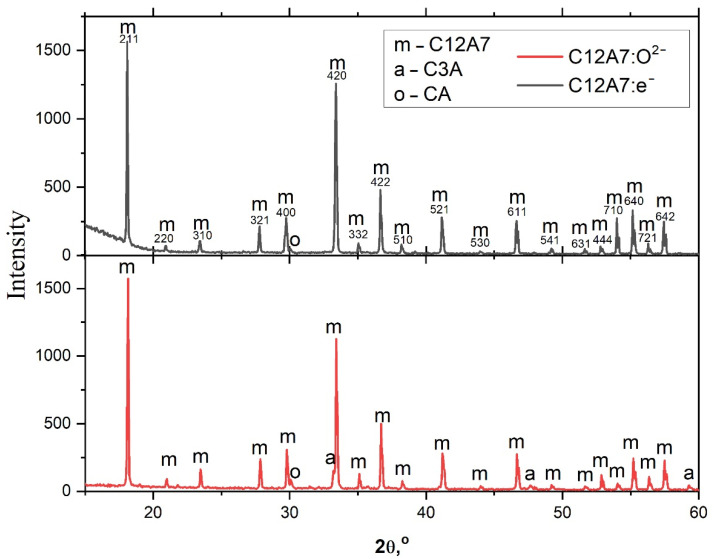
XRD patterns of initial samples C12A7:e^−^ (**upper**) and C12A7:O^2−^ (**lower**).

To confirm the phase composition and structure of the obtained material, the samples were analyzed by XRD and electron microscopy. According to the XRD data (Figure 2), the phase composition of the samples corresponds to the expected results. The main phase is mayenite (up to 90–95% of the target phase), presumably containing various anions (mainly O^2−^ in the case of ordinary mayenite and e^−^ in the case of electride). The obtained samples are well crystallized, with crystallite sizes over 150 nm. Insignificant impurities of calcium aluminates Ca_3_Al_2_O_6_ (C3A) and CaAl_2_O_4_ (CA) are also observed in the composition of materials. The presence of impurities does not significantly affect the properties under study because these phases are also well crystallized and segregated in local areas of the material volume. A similar phase composition at synthesis temperatures and the stoichiometry of the elements have already been noted earlier in the literature [13,20]. The unit cell parameter of mayenite C12A7:O^2−^ is smaller (11.998 ± 0.001 Å) than its electride state C12A7:e^−^ (12.004 ± 0.003 Å), which is associated, in the case of an electride, with the formation of oxygen vacancies and weaker interaction electrons with a cationic sublattice, as a result of which the unit cell of the crystal slightly increases [22,23].

**Figure 3:**
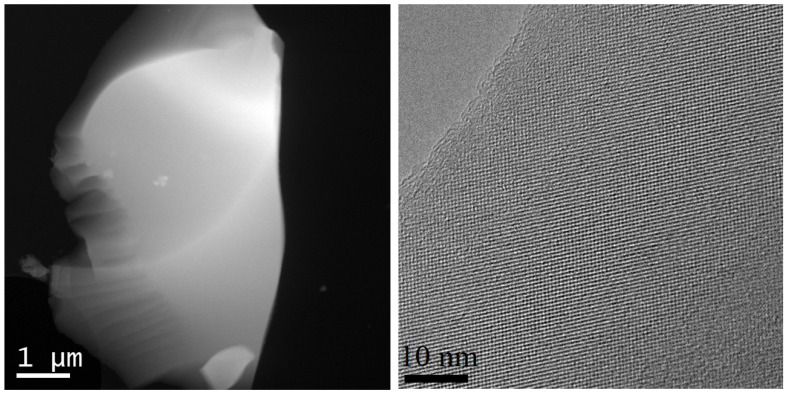
Morphology and microstructure of particles on the example of a sample C12A7:e^−^.

In Figure 3, the particles’ microstructure of the investigated samples of electride C12A7:e^−^ and mayenite C12A7:O^2−^ is presented. The micrograph clearly shows a characteristic cubic crystal lattice with a small number of defects, as well as small formations of an amorphous substance on the particle surface. The particles in the samples have a flat irregular shape, as well as a rather large size (0.5–5 μm).

### 3.2. EPR Studies

Regardless of the mayenite synthesis method during the formation of the electride state in it, the C12A7:e^−^ material, along with conduction electrons, always has a significant concentration of paramagnetic centers (the s-called F^+^-like centers), which give a characteristic signal with g = 1.994 in the EPR spectra [12,24,25,26,27]. The concentration of these centers does not exceed 2·10^19^ g^−1^, correlates with conductivity, and is the same for mono- and polycrystalline electride samples [24].

**Figure 4:**
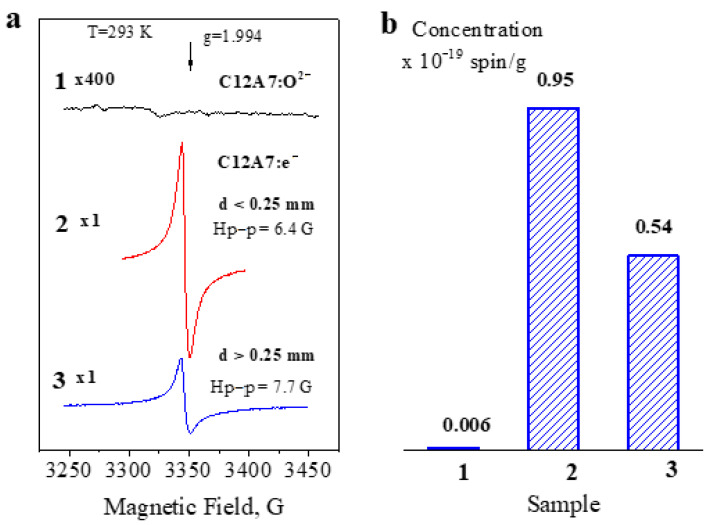
(**a**) EPR spectra of sample C12A7:O^2−^ (1) and sample C12A7:e^−^ after its grinding in an agate mortar with separation of fractions d < 0.25 mm (2) and d > 0.25 mm (3). (**b**) Concentration of paramagnetic centers in these samples.

Figure 4a shows typical EPR spectra of the samples used in this work. It is seen that the appearance of these centers is not observed in the C12A7:O^2−^ sample. At the same time, C12A7:e^−^ samples are characterized by an intense signal from F^+^-like centers (g = 1.994, H_p–p_ = 6.4 G), whose concentration is about 1·10^19^ g^−1^. It should be noted that spectrum two shown in Figure 4a for the d < 0.25 mm fraction of this sample, which is well described by the Lorentzian line shape. At the same time, in the EPR spectra of larger (d > 0.25 mm) fractions of the same sample (Figure 4a, spectrum 3), a significant distortion of the shape and the appearance of asymmetry are observed due to a significant contribution from the Dyson line shape associated with the present skin layers in the conductive samples. It should be noted that the procedure for grinding the massive (Figure 1b) electride C12A7:e^−^ is necessary for the measurement of the samples by the EPR method. The registration of the spectra with its massive samples, with a size of more than 1–2 mm, is practically impossible due to the very high absorption of microwave power and the impossibility of adjusting the spectrometer.

### 3.3. Raman Studies

**Figure 5:**
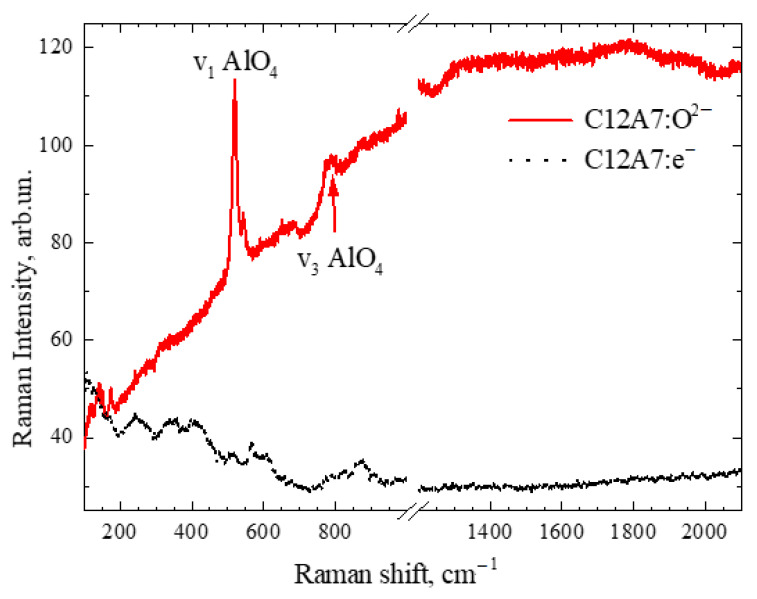
Raman spectra of C12A7:O^2−^ (**red**) and C12A7:e^−^ (**black**).

Figure 5 shows the Raman spectra of the mayenite (C12A7:O^2−^, white sample) and electride (C12A7:e^−^, black sample).

The spectra of the white sample contain the most intense peaks from mayenite C12A7 [28,29] (Figure 5). These are v_1_–AlO_4_ (517–520 cm^−1^) and v_3_–AlO_4_ (777–784 cm^−1^) modes. In the black sample, these peaks do not appear clearly, presumably for two reasons. The first reason for this is that this sample may contain layers of opaque coatings of carbon on the surface (as an impurity from a graphite crucible), due to which light does not reach the mayenite volume. The second reason for this is that the sample is conductive so the light hardly penetrates it, but it is reflected due to plasma scattering. However, in the Raman spectra of both samples, D (~1350 cm^−1^) and G (~1580 cm^−1^) peaks from amorphous carbon or from graphite are not observed. As such, it is assumed that the absence of the expected v_1_ and v_3_ peaks is due to the second reason. Previously, it was also indicated in the literature that the spectrum may contain a peak at 186 cm^−1^, which grows with an increase in the concentration of free electrons [30]; however, in our samples, this does not manifest itself explicitly, and only weak features are observed at ~140 cm^−1^.

### 3.4. FTIR Spectroscopy

**Figure 6:**
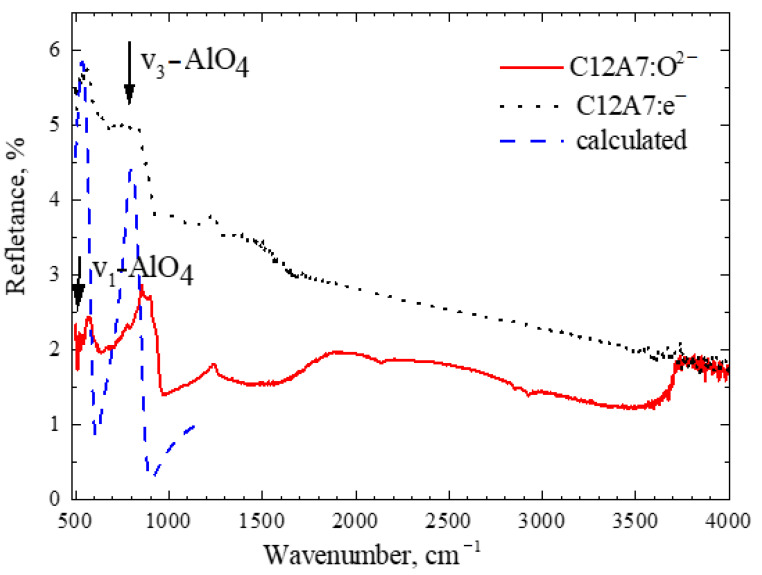
Experimental FTIR reflectance spectra of the samples (**black** and **red** curves) and reflectance calculated in the Drude–Lorentz model (**blue** curve, values divided by 10).

The FTIR reflection spectra of both samples are shown in Figure 6. The arrows indicate the frequencies of the v_1_–AlO_4_ (520 cm^−1^) and v_3_–AlO_4_ (780 cm^−1^) modes, which were observed in the Raman spectra. In contrast to the absorption spectra (in which the absorption peak corresponds to the vibrational corresponding frequency), the interpretation of the reflection spectra is difficult. Therefore, a simulation of reflection from a semi-infinite medium at a normal incidence was carried out, in which the complex index of the material was described by the Drude–Lorentz model.

In the Drude–Lorentz model, the dependence of the complex permittivity *ε* n the frequency *υ* is calculated as:

(1) $$\varepsilon \left(\nu \right)=\varepsilon_{\infty }(1+\sum_{i=1}^{K}\frac{\Omega_{pi}^{2}}{\nu_{i}^{2}-\nu^{2}-i\nu \Gamma_{i}})$$

here $\varepsilon_{\infty }$ is the permittivity for frequencies much higher than the vibrational frequencies of atoms; *ν_i_* are the frequencies of oscillations of bound charges; $\Gamma_{i}$ is the damping of oscillations of bound charges; and $\Omega_{pi}=\frac{e_{i}}{2\pi c}\sqrt{\frac{4\pi N_{i}}{3\cdot m_{i}\cdot \varepsilon_{\infty }}}$ are plasma frequencies of bound charges with effective mass *m_i_*, effective charge *q_i_*, and concentration *N_i_*.

The dependence of the complex refractive index *N*(*υ*) is calculated as:

(2) $$N\left(\nu \right)=\sqrt{\varepsilon \left(\nu \right)}=n\left(\nu \right)+ik\left(\nu \right)$$

Then, according to the Fresnel formula, the value of the intensity reflection coefficient is calculated.

(3) $$R\left(\nu \right)=\frac{1-N\left(\nu \right)}{1+N\left(\nu \right)}\cdot {\left(\frac{1-N\left(\nu \right)}{1+N\left(\nu \right)}\right)}^{*}$$

The vibration frequencies of the bound charges corresponded to the frequencies of the v_1_–AlO_4_ (520 cm^−1^) and v_3_–AlO_4_ (780 cm^−1^) modes, the damping was 40 and 60 cm^−1^, respectively. The plasma frequencies of both modes were calculated based on the concentration of bound charges 5·10^22^ cm^−3^, the effective charge was taken to be equal to the electron charge, the effective mass corresponded to the mass of atomic oxygen, and the dielectric constant at high frequencies was taken equal to five. The reflection spectrum for normal incidence was calculated in the range of 500–1100 cm^−1^.

It can be seen that, in general terms, the calculated reflection spectrum repeats the features observed in the experimental spectra. Slightly above the frequency of each mode, an increase in reflection is first observed, followed by a dip. It can also be seen that the magnitude of the experimental reflection coefficient is approximately an order of magnitude less than the calculated one (the values of the latter are divided by 10 for convenience of perception). This is because the surface of the samples is not specular, and diffuse scattering predominates, rather than specular reflection. The decrease in reflection with a decrease in wavelength (respectively, with an increase in wave number) can be caused by the fact that short-wave radiation experiences more diffuse scattering. Some differences between the calculated and experimental spectra are also related to the fact that the experimental spectra were recorded at an angle of incidence of 45 degrees, and not for normal incidence. For example, one can see that the experimental peak of C12A7:O^2−^ (red curve in Figure 6) between 800 and 1000cm^−1^ is shifted and wider compared to the calculated peak (blue curve in Figure 6). It should be noted that the model used is very simple and does not reflect all the features of the experimental spectrum. This is primarily due to the fact that the primitive cell of the studied 12CaO·7Al_2_O_3_ material contains 59 atoms. This means that the number of phonon modes is 59 × 3 = 177. Note that 177 − 3 = 174 are optical phonon modes. Only two (Raman active) modes were used for modeling, but other modes can be active in FTIR absorption. This can lead to a broadening of and a shift in the band experimentally observed in the reflection.

In general, it can be said that the v_1_–AlO_4_ (520 cm^−1^) and v_3_–AlO_4_ (780 cm^−1^) modes, which were observed in the Raman spectra, appear in the reflection spectra. Similar features were also observed in the absorption and transmission spectra of the infrared radiation in mayenites [29,31,32]. In addition, in the experimental spectra of both samples, there were features with a frequency between 1000 and 1500 cm^−1^. This may be a contribution from the vertical vibrations of Al–O [31] (Al–O vertical vibrations). The features observed in the spectrum of mayenite with a frequency of more than 3600 cm^−1^ may be due to the contribution of O–H vibrations [29].

### 3.5. Electrophysical Studies

**Figure 7:**
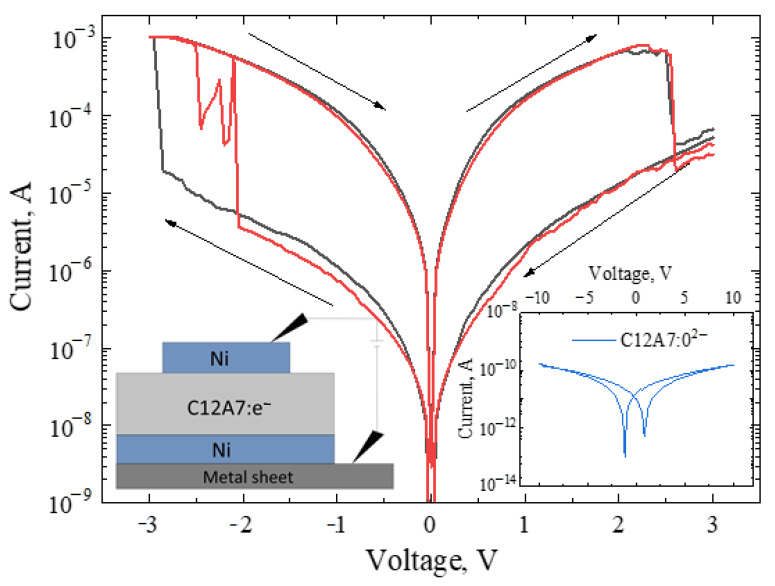
I–V curve of electride C12A7:e^−^ and mayenite C12A7:O^2−^ (inset) at room temperature.

Figure 7 shows the current–voltage characteristics (I–V curves) of electride C12A7:e^−^. The initial sweep voltage for the electride was 3 V. From 3 V to −3 V, the state of the HRS (lower branch of the I–V curve characteristic) was recorded during the sweep. The switching voltage from the HRS to the LRS (turn on) varied from −2 V to −3 V. After reaching the switching point, the state was transferred to an LRS, and the LRS is observed in the reverse direction of the sweep. The switching voltage from the LRS to the HRS was 2.5 V. Monocrystalline and polycrystalline C12A7:e^−^ are characterized by several resistive states [13,14]. This phenomenon is called the memristor effect and indicates the prospects for using mayenite-type electride as the main dielectric in ReRAM devices [14]. The I–V curve for oxygen-containing mayenite C12A7:O^2−^ is shown in the inset in Figure 7, and the measurement range was from 10 to −10 V. According to the obtained dependence of current on voltage, in the normal state, mayenite does not have memristor properties. 

The electride material is a polycrystal with two types of anions: oxygen and an electron. When a negative voltage is applied to the top electrode for the first time, oxygen anions migrate, thereby creating a concentration gradient of oxygen ions in the electride. An electride with an oxygen gradient in the structure is similar to bilayer structures [14,33]. Thus, the studied mayenite-based structure can be used in memristors. Memristor structures are characterized by jump-like switching on current–voltage characteristics. In Figure 7, one can see that, at the moment of transition between the different states, a current jump occurs, which is called a soft reversible breakdown. The electride-based sample requires a filament-forming process called electroforming, which was conducted by bias alternation from −3 V to 3 V before the cycling shown in Figure 7.

**Figure 8:**
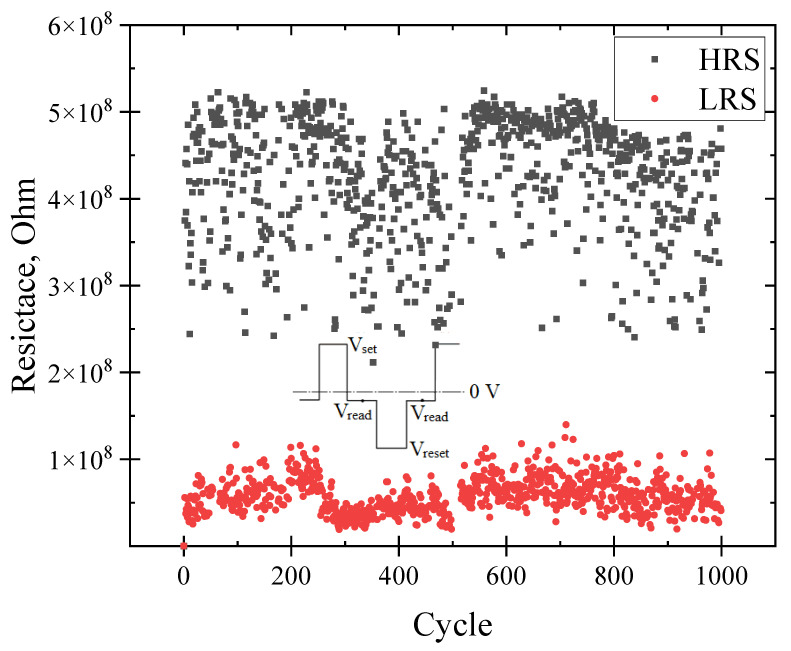
Endurance of memristors (resistance of memristors in HRS and LRS states under 1000 cycles).

Figure 8 shows 1000 cycles of electrode re-cycling. The reading voltage was −1.5 V. The cycle time was 100 ms. The switching pulse time was 10 ms. V_set_/V_reset_ was +5/−5 V, respectively. The cycle measurement scheme is shown in Figure 3. When looking at Figure 8, two states can be distinguished, which were kept for 1000 cycles. 

**Figure 9:**
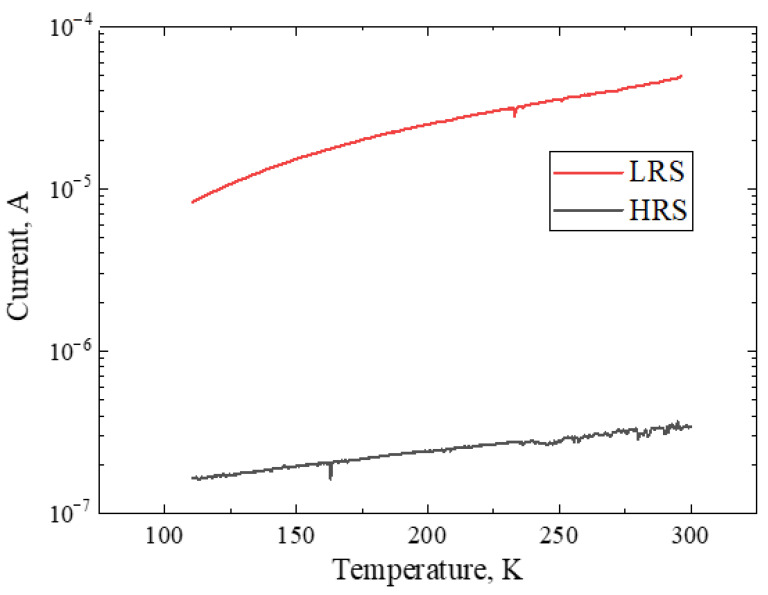
Current dependence on temperature in HRS (**black**) and LRS (**red**) states for C12A7:e^−^.

Figure 9 shows a general view of the temperature dependences of the conductivity of the C12A7:e^−^ sample in the HRS and LRS states. The read voltage for the current readout was 0.5 V. The behavior of the current in the HRS state when the temperature change had a similar trend with the behavior of the current in the LRS state, i.e., the current increased as the temperature increased. The decrease in conductivity with decreasing temperature indicates a semiconductor type of conductivity. However, monocrystalline electride has mainly metallic conductivity [30,34]. This can mean that there are two types of anions in the C12A7:e^−^ sample, i.e., oxygen ions O^2−^ and electrons e^−^. Based on this, we can conclude that in the electrode, to create the possibility of resistive switching, it is sufficient to partially replace the O^2−^ anions with e^−^ electrons.

To study the mechanism of charge transport in an electride, the Arrhenius rule of rate theory and the Hurd quantum-tunnelling model were used [35]. The Arrhenius rule can show that over-barrier charge emissions or thermally facilitated tunnelling predominate in the material. This rule of rate is as follows:

J ~ exp(−U/kT)(4)

where U is the activation energy and k is the Boltzmann constant.

Hurd’s model involves charge tunnelling through the barrier. The generalized current density according to Hurd’s model is presented as [35]:

J ~ exp(T/T_b_) (5)

where T_b_ is Berthelot temperature.

**Figure 10:**
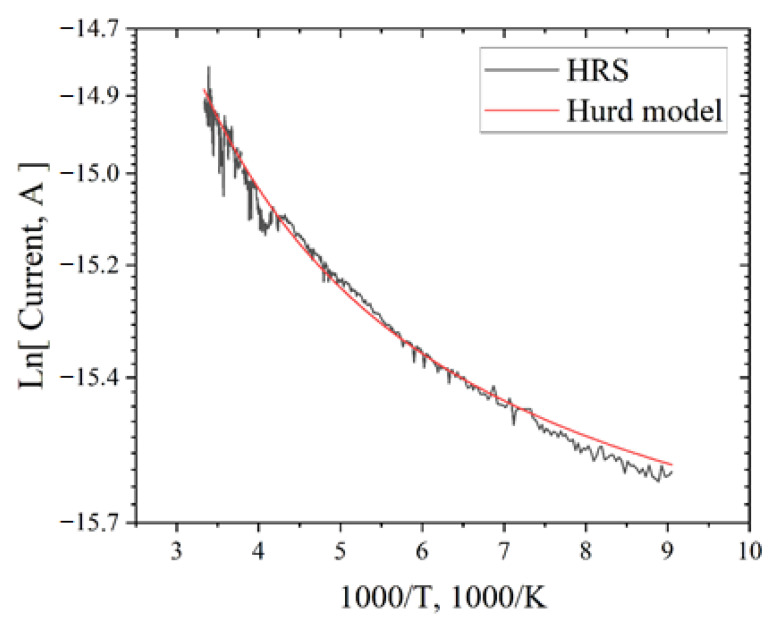
Dependence of current on temperature for the HRS state (**black**) and approximation curve by the Hurd model (**red**) for C12A7:e^−^.

Figure 10 shows the experimental and calculated curves of the temperature dependence of conductivity in the HRS state within the Hurd quantum-tunnelling model. The Arrhenius rule in the HRS state did not adequately approximate the experimental data. When approximated by the Hurd model, the Berthelot temperature was determined, which in energy units is 22 meV (or 255 K). The approximation data show that charge tunnelling between traps dominates in the HRS state.

**Figure 11:**
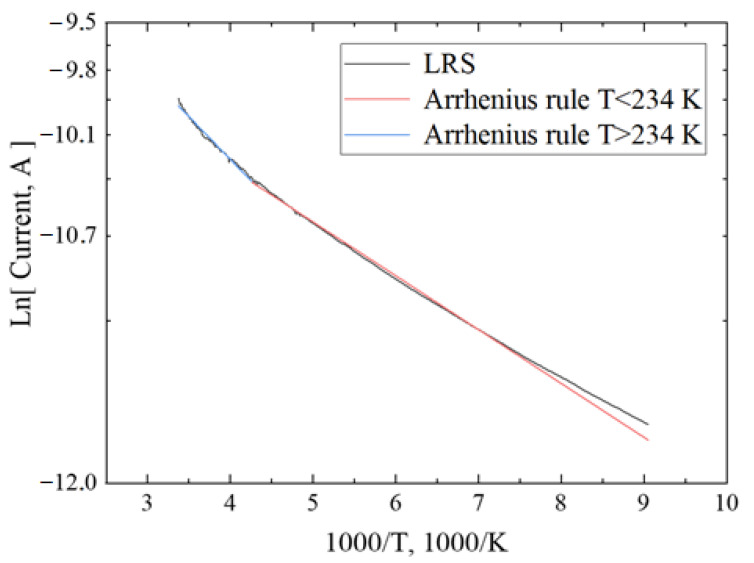
Dependence of current on temperature for the LRS state (**black**) and two approximation curves (before 234 K **red** and after 234 K **blue**) by the Arrhenius model.

Figure 11 shows the experimental and calculated curves of the temperature dependence of conductivity in the LRS state, approximated by the Arrhenius rule. The approximation of the LRS state showed that there is a point of change in the activation energy, by an analogy of the conductivity in semiconductors [36] from 234 K to 100 K. This assumption may exist in the framework of the Arrhenius rule. The activation energy for the low-temperature region is 25.5 meV (or 295 K). For the high-temperature region, the activation energy is 40.6 meV (or 467.9 K). It can be concluded that the over-barrier emission from traps dominates in the LRS state.

Different resistance states have different conduction mechanisms. It is known that a monocrystalline electride is capable of transitioning from a metallic state to a metal-semiconductor state, depending on the concentration of free electrons [36]. It is possible that when switching between states, the electron concentration changes, which leads to a change in the transport mechanism.

**Figure 12:**
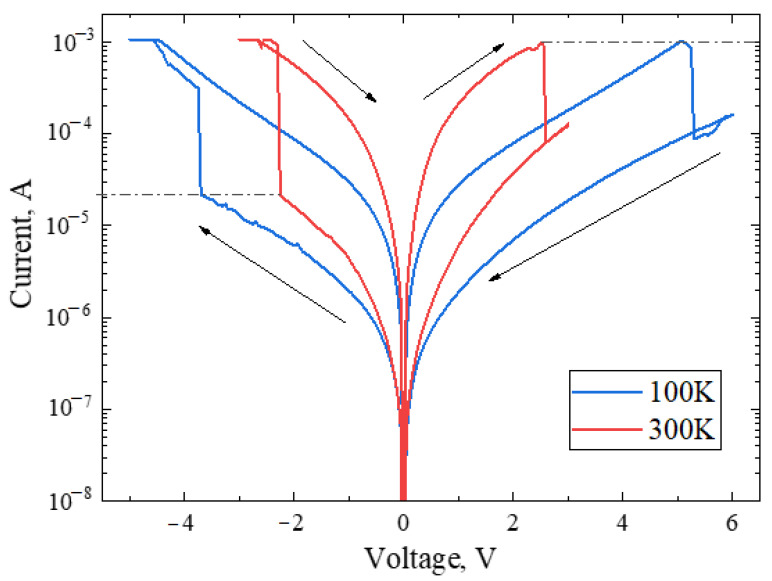
I–V curves the sample C12A7:e^−^ at the temperatures of 100 K (**blue**) and 300 K (**red**).

I–V curve characteristics at the extreme points of measurement at 100 and 300 K are shown in Figure 12. From the analysis of the curves, we can conclude that with a decrease in temperature by 200 K, both the ON and OFF voltage (i.e., biases required for the transition to LRS and HRS states, correspondingly) increased by 2–3 V, while the current at the moment of switching remained the same. Probably, switching between resistive states depends on local heating in regions where the concentration of anion–electrons in mayenite is higher.

## 4. Conclusions

The studies of the I–V curve characteristics of a material based on polycrystalline mayenite C12A7:e^−^ showed the presence of bipolar resistive switching. A detailed analysis of the temperature characteristics revealed that the C12A7:e^−^ sample has semiconductor conductivity. This may indicate the presence of two types of anions in the polycrystalline electrode. The study of the mechanisms of the charge transport of memristors based on polycrystalline C12A7:e^−^ showed that the LRS and HRS states differ in the mechanism of conduction. This effect is associated with the transition from the metallic state to the semiconductor state, depending on the concentration of free electrons. Presumably, Hurd’s quantum tunnelling predominates in the HRS state, while the thermal emission of electrons from traps predominates in the LRS state. When analyzing the I–V characteristics at different temperatures, it was revealed that switching between states occurs when a certain current threshold is reached, and not a voltage threshold.

## Data Availability

The data presented in this study are available on request from the corresponding authors.
